# Does Minimally Invasive Transsacral Fixation Provide Anterior Column Support in Adult Scoliosis?

**DOI:** 10.1007/s11999-013-3335-6

**Published:** 2013-11-06

**Authors:** Neel Anand, Eli M. Baron, Babak Khandehroo

**Affiliations:** 1 Spine Center, Cedars-Sinai Medical Center, 444 S San Vicente Blvd, Suite 800, Los Angeles, CA 90048 USA; 2 Department of Neurosurgery, Cedars Sinai Medical Center, Los Angeles, CA USA

## Abstract

**Background:**

Spinal fusion to the sacrum, especially in the setting of deformity and long constructs, is associated with high complication and pseudarthrosis rates. Transsacral discectomy, fusion, and fixation is a minimally invasive spine surgery technique that provides very rigid fixation. To date, this has been minimally studied in the setting of spinal deformity correction.

**Questions/purposes:**

We determined (1) the fusion rate of long-segment arthrodeses, (2) heath-related quality-of-life (HRQOL) outcomes (VAS pain score, Oswestry Disability Index [ODI], SF-36), and (3) the common complications and their frequency in adult patients with scoliosis undergoing transsacral fixation without supplemental pelvic fixation.

**Methods:**

Between April 2007 and May 2011, 92 patients had fusion of three or more segments extending to the sacrum for spinal deformity. Transsacral L5-S1 fusion without supplemental pelvic fixation was performed in 56 patients. Of these, 46 with complete data points and a minimum of 2 years of followup (mean, 48 months; range, 24–72 months; 18% of patients lost to followup) were included in this study. Nineteen of the 46 (41%) had fusions extending above the thoracolumbar junction, with one patient having fusion into the proximal thoracic spine (T3-S1). General indications for the use of transsacral fixation were situations where the fusion needed to be extended to the sacrum, such as spondylolisthesis, prior laminectomy, stenosis, oblique take-off, and disc degeneration at L5-S1. Contraindications included anatomic variations in the sacrum, vascular anomalies, prior intrapelvic surgery, and rectal fistulas or abscesses. Fusion rates were assessed by full-length radiographs and CT scanning. HRQOL data, including VAS pain score, ODI, and SF-36 scores, were assessed at all pre- and postoperative visits. Intraoperative and postoperative complications were noted.

**Results:**

Forty-one of 46 patients (89%) developed a solid fusion at L5-S1. There were significant improvements in all HRQOL parameters. Eight patients had complications related to the transsacral fusion, including five pseudarthroses and three superficial wound dehiscences. Three patients underwent revision surgery with iliac fixation. There were no bowel injuries, sacral hematomas, or sacral fractures.

**Conclusions:**

Transsacral fixation/fusion may allow for safe lumbosacral fusion without iliac fixation in the setting of long-segment constructs in carefully selected patients. This study was retrospective and suffered from some loss to followup; future prospective trials are called for to compare this technique to other, more established approaches.

**Level of Evidence:**

Level IV, therapeutic study. See Instructions for Authors for a complete description of levels of evidence.

## Introduction

Spinal fusion procedures are performed for correction of a wide spectrum of spinal disorders. Traditionally these are performed through open surgical approaches. Open surgery can result in approach-related morbidity and complications, including muscle dysfunction, infection, and blood loss [21, 23, 27, 29–31, 38, 41, 42, 47]. In recent years, however, a better understanding of surgical anatomy, combined with advanced technologies and newer techniques, has allowed many spine conditions to be treated in a less invasive fashion. These techniques have allowed the surgeon to move toward using smaller incisions with less tissue trauma when performing corrective procedures on the spine [19, 39]. Less invasive approaches may result in decreased postoperative pain, reduced postoperative medication usage, shorter hospitalizations, quicker return to daily activities, and diminished healthcare costs compared with traditional approaches [3, 6, 7].

Over the last several decades, a number of approaches have been developed to lumbosacral interbody fusion. These include anterior lumbar interbody fusion (ALIF) [15], posterior lumbar interbody fusion [16], and transforaminal lumbar interbody fusion (TLIF) [26, 36]. Subsequently, less invasive variants of the ALIF and TLIF have been developed [28, 45, 50]. The combination of anatomic limitations and morbidity of approaches for fusion of the lumbosacral disc space, the high L5-S1 pseudoarthrosis rates at the bottom of a long construct, and the poor anatomic configuration of the S1 pedicle for screw fixation has resulted in the development of newer reproaches for achieving L5-S1 interbody fusion. The percutaneous, paracoccygeal presacral approach technique, initially described by Cragg et al. [17], addresses the lumbosacral disc space along the longitudinal axis of the sacrum. This is performed minimally invasively through the presacral space using a 3-cm incision. It does not require an abdominal approach nor does it require mobilization or retraction of the vasculature or intraabdominal contents [17, 37, 49]. Because of this and the minimal tissue disruption associated with the presacral approach, this technique may reduce the risk of approach-related complications and morbidities associated with traditional approaches to the L5-S1 disc space. To date, this approach has been minimally studied in the setting of spinal deformity correction.

We therefore determined (1) the fusion rate of long-segment arthrodeses, (2) heath-related quality-of-life (HRQOL) outcomes (VAS pain score, Oswestry Disability Index [ODI], SF-36), and (3) the common complications and their frequency in adult patients with scoliosis undergoing transsacral fixation without supplemental pelvic fixation.

## Patients and Methods

### Patient Selection

Data for this study were obtained through a retrospective chart review with institutional review board approval. Outcome data were prospectively collected at each visit through self-administered patient questionnaires. All surgeries were performed by the senior spine surgeon (NA) at a single tertiary academic center between April 2007 and May 2011.

A database review of surgical cases performed by the senior author revealed 92 patients who underwent a fusion of three or more levels that extended across the lumbosacral junction; of those, 56 underwent transsacral (L5-S1) fusion at the bottom of a long construct for spinal deformity, and 46 (82%) had complete data points at a minimum followup of 2 years and were included in this study (Table 1). Of the 10 patients with missing data points, seven had their last followup at 18 months, two at 1 year, and one at 6 months. There were 20 men and 26 women with a mean age of 67 years (range, 22–81 years), with the majority (n = 38) in their 60s and 70s. The mean number of levels operated on was 5.6 (range, 3–15). Nineteen of the 46 included patients (41%) had fusion extending above the thoracolumbar junction, with one having fusion into the proximal thoracic spine (T3-S1). Deformities included degenerative scoliosis (n = 33), idiopathic scoliosis (n = 9), and iatrogenic scoliosis (n = 4). During the period in question, indications for performing transsacral fusion at L5-S1 were situations where the fusion needed to be extended to the sacrum and there were no contraindications to the presacral approach, including the presence of L5-S1 spondylolisthesis, prior L5-S1 laminectomy, L5-S1 stenosis, oblique take-off at L5-S1, and L5-S1 disc degeneration [14]. Contraindications included anatomic variations in the sacrum, vascular anomalies, prior intrapelvic surgery, and rectal fistulas or abscesses.

**Table 1:** Demographic data

Diagnosis	Number of patients			Mean age (years)
	Total	Male	Female	
Degenerative scoliosis	33	17	16	69.3
Idiopathic scoliosis	9	1	8	60
Iatrogenic scoliosis	4	2	2	65.7
Total	46	20	26	67

All patients underwent circumferential minimally invasive deformity correction and fusion using all or a combination of three minimally invasive surgical techniques: segmental multilevel percutaneous pedicle screw fixation, correction, and fusion; lateral transpsoas discectomy and interbody fusion; and transsacral fixation and fusion (AxiaLIF^®^) (TranS1 Inc, Wilmington, NC, USA). All patients had participated in extensive nonoperative therapies without relief of their symptoms before being considered for surgery. No patient underwent supplemental iliac fixation. The minimum followup was 24 months (mean, 48 months; range, 24–72 months); loss to followup was 18% (10 of 56 patients).

### Surgical Technique

**Fig. 1A–B:**
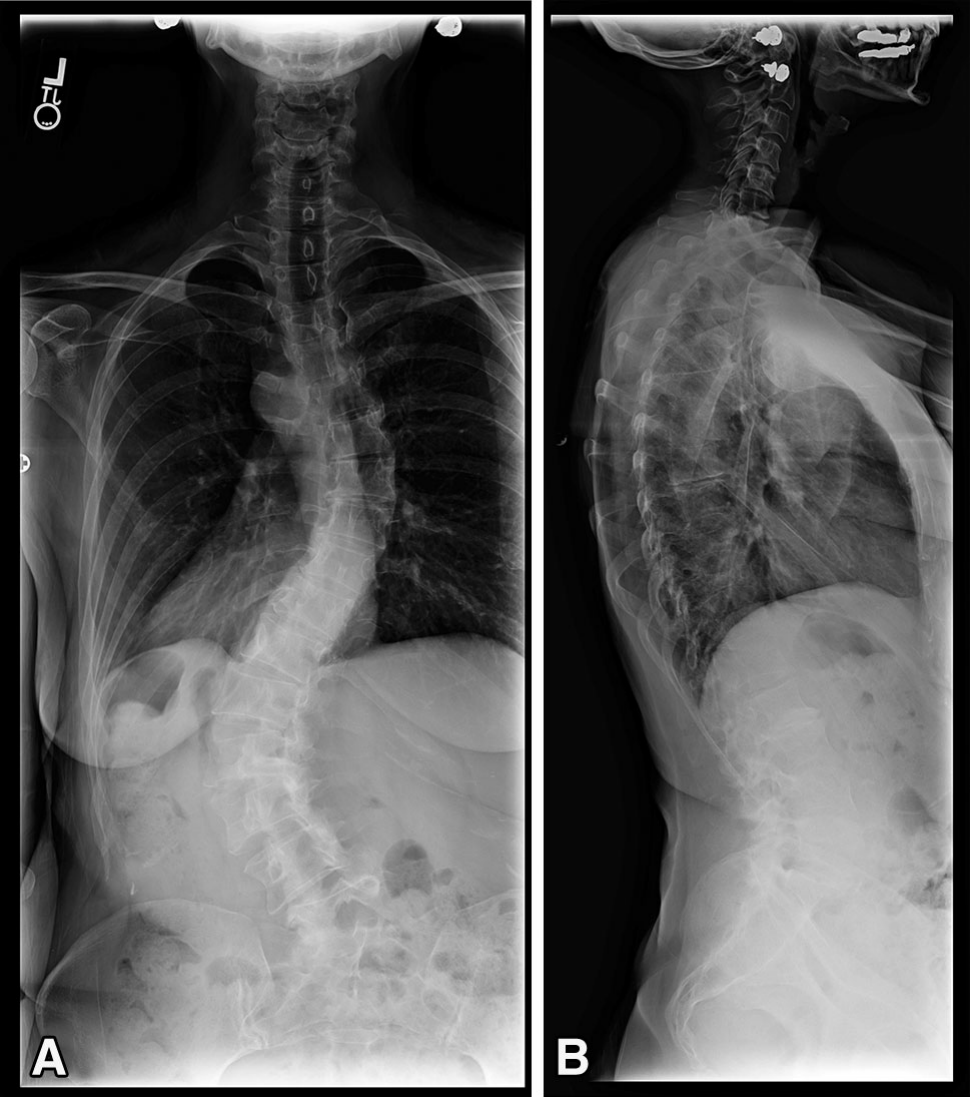
These 36-inch (**A**) AP and (**B**) lateral standing radiographs show the spine of a 53-year-old woman complaining of back and leg pain. Workup revealed her to have adult idiopathic scoliosis. She has a left curve from T10-L4 measuring 58°, a right curve from T5-T10 of 34°, and a fractional curve of L4-S1 measuring 33°.

As described above, three techniques were used for the circumferential minimally invasive correction of spinal deformity (Fig. 1). Transpsoas discectomy and fusion, as well as minimally invasive posterior spinal instrumentation and fusion for spinal deformity correction, have been described in detail elsewhere [6, 7]. Technical aspects of transsacral discectomy, fusion, and fixation have also been described elsewhere [2]. In terms of interbody grafting at L5-S1, we used 2.1 mg rhBMP-2 absorbable collagen sponge (Medtronic, Inc, Minneapolis, MN, USA) in the disc space in addition to Grafton^®^ Putty demineralized bone matrix (Medtronic, Inc). Supplemental minimally invasive posterior pedicle screw fixation was always used and a posterolateral facet fusion using 1 to 1.5 mg rhBMP-2 absorbable collagen sponge in each pars-facet complex was performed [1, 7].

All patients underwent preoperative plain radiography that included the entire sacrum and coccyx. Anatomic variations of the sacrum such as a hook-shaped sacrum or a very flat sacrum may make the appropriate trajectory for placement of the transsacral fixation screw very difficult to near impossible. This mandates appropriate preoperative templating and planning. Additionally, MRI of the lumbar spine and the pelvis were performed to assess vascular anatomy, as it is critical to rule out any aberrant midline blood vessels in the region of S1-S2. MRI was also performed to make sure there was an adequate fat pad in the presacral space. Adhesions in this region were a contraindication to this procedure [12]. If osteoporosis was suspected, a dual-energy x-ray absorptiometry study was obtained. A T-score of less than −2.5 of the femoral neck contraindicated this technique. If significant coronal deformity was present at the fractional lumbosacral curve, then correction of this deformity with the screws and rods was first achieved before fixation of the L5-S1 segment using this technique.

Postoperatively patients were allowed to ambulate on Postoperative Day 1. An occlusive dressing was kept over the transsacral incision for a week. Bracing was not routinely used.

### Outcomes

Postoperative visits were scheduled at 6 weeks, 3 months, 6 months, 1 year, 2 years, and yearly thereafter. Fusion was assessed at 1 year using radiographs, including flexion/extension films of the lumbar spine. CT scan was routinely done between 12 and 18 months for assessment of fusion. The presence of bridging bone in and around interbody grafts was looked for to confirm fusion, in addition to fused facets on sagittal and coronal reconstructions and lack of periimplant lucencies. CT scans were available for 44 of the 46 patients; the other two, who declined a CT scan, had solid fusions evident on plain radiographs and no clinical symptoms. Clinical outcome data, including pain score on a 100-point VAS, ODI, and SF-36 score, were collected at each visit through self-administered patient questionnaires. Intraoperative and postoperative complications were noted.

### Statistics

Unpaired t-tests were used to calculate significance of postoperative clinical outcomes, where a p value of less than 0.05 was considered the threshold for significance. We performed statistical analyses using Microsoft^®^ Excel^®^ (Microsoft Corp, Redmond, WA, USA).

## Results

**Fig. 2A–B:**
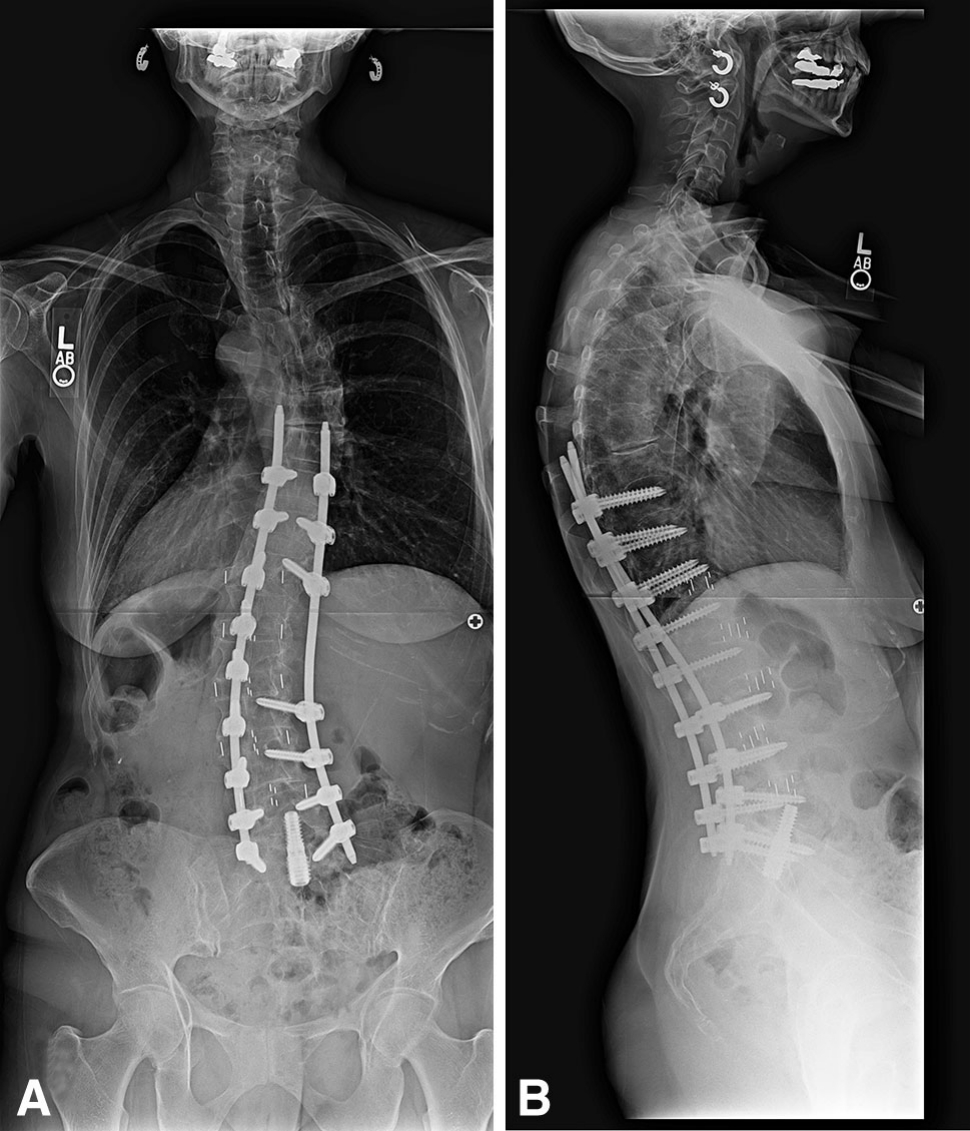
These 36-inch (**A**) AP and (**B**) lateral standing radiographs show the spine of the patient in Figure 1 at 3 years after lateral transpsoas discectomy and interbody fusion, percutaneous pedicle screw and rod placement, and L5-S1 transsacral discectomy and interbody fusion. A solid fusion was achieved at L5-S1 without iliac fixation.

**Fig. 3A–B:**
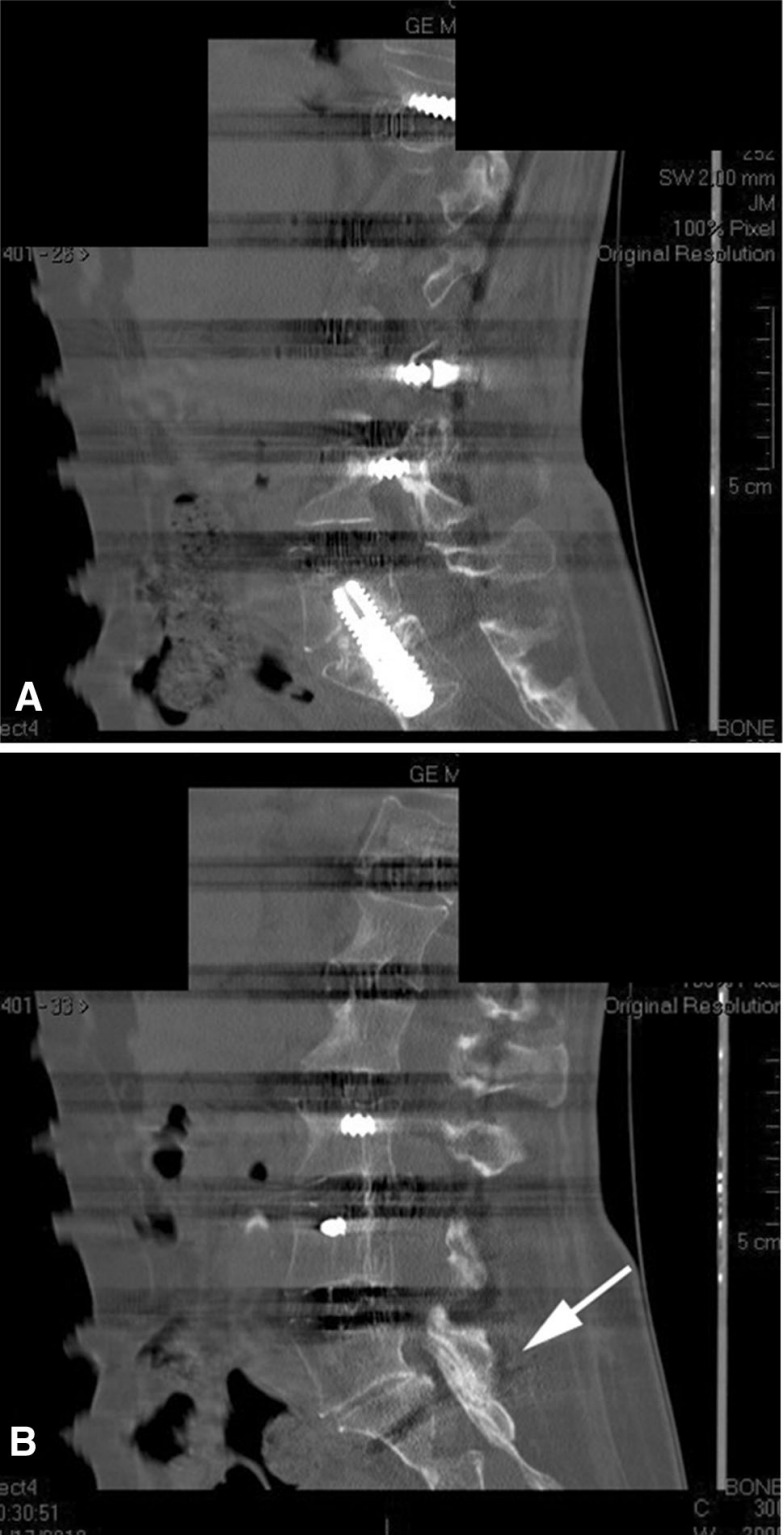
Sagittal CT reconstructions 1 year after minimally invasive deformity reconstruction are shown. (**A**) A midsagittal cut shows some anterior interbody bone material. (**B**) An image through the L5-S1 facet shows a solid facet fusion (arrow) after use of BMP, local bone, and demineralized bone matrix for fusion.

Forty-one of 46 patients developed a fusion at L5-S1 (Figs. 2, 3), for an overall fusion rate of 89%. Five of the 46 patients (10.8%) developed an L5-S1 pseudarthrosis. Two were in the setting of late-onset infection; one occurred after 1 year postoperatively and the other at 18 months postoperatively. The first patient was revised with removal of the implant, ALIF, and iliac screw fixation and the second patient with iliac screws and posterior reinstrumentation. Two patients had an L5-S1 nonunion with sacral pedicle screw loosening. These patients had a posterior revision with new S1 pedicle screws and bilateral iliac screws. The fifth patient had an asymptomatic pseudarthrosis. CT scan after revision surgery confirmed fusion in the four patients and the other patient continues to have an asymptomatic pseudarthrosis. There were no cases of transsacral screw misplacement.

There were improvements in all clinical outcome parameters at all postoperative visits (Table 2). The 10 patients lost to 2-year followup all had improving outcomes at their last visit and radiographs were showing progressive fusion.

**Table 2:** Clinical and functional outcomes

Variable	Mean score (points)							p value
	Preoperative	6 weeks	6 months	12 months	24 months	36 months	> 36 months	
VAS	65.8	37	31	33	34	30	28	< 0.001
ODI	47.6	43.6	33.5	33	33	23	21.8	< 0.001
SF-36	40	51.7	52	57	62	69	70	0.01

ODI = Oswestry Disability Index.

Eighteen postoperative complications were noted in 17 patients (Table 3); eight were directly concerning the transsacral fixation procedure, and these occurred in eight patients. All other complications were related to other techniques used in minimally invasive correction of spinal deformity and have recently been reported elsewhere [4]. Three patients had a superficial sacral wound dehiscence and subsequently underwent débridement of their incisions followed by secondary closure. There were no intraoperative complications. There were no bowel injuries, sacral hematomas, or sacral fractures.

**Table 3:** Complications of transsacral fixation

Complication	Number of patients	Intervention
Superficial wound dehiscence	3	Local wound care
Pseudarthrosis (L5-S1)	5	
Late infection: loose transsacral screw	1	Removal of transsacral screw, ALIF, posterior extension to ilium
Late infection: loose sacral screw	1	Reinstrumentation S1 screws and extension to ilium
Noninfected: loose sacral screw	2	Posterior reinstrumentation S1 screws and extension to ilium
Asymptomatic	1	Continued observation
Total number of transsacral complications	8	
Total number of patients with complications	8	

ALIF = anterior lumbar interbody fusion.

## Discussion

In recent years, a better understanding of surgical anatomy, combined with advanced technologies and newer techniques, has allowed many spine conditions to be treated in a less invasive fashion than traditional open methods. In this study, we were interested in evaluating a less invasive approach to arthrodesis at the lumbosacral junction at the bottom of a long-segment fusion; this approach does not require an abdominal approach nor does it require mobilization or retraction of the vasculature or intraabdominal contents. With this approach, our overall fusion rate was 89%, HRQOL data improved after surgery, and our overall complication rate was 39%, with 17% of the complications being directly related to transsacral fixation.

This study had a number of limitations. This was a relatively small series of patients studied without a control group. Given the small sample size, rare complications would be potentially missed. Additionally, this was a retrospective review, raising the possibility of selection bias affecting the application of the technique. Finally, 18% of our patients were lost to followup before 2 years (10 of 56 patients); it is possible that some of these patients had complications or failures of treatment but did not return to our center for evaluation. As such, our results should be considered a best-case scenario with this technique.

The frequency of fusion we observed compares well with the fusion rates to the sacrum reported in other studies, especially in the setting of spinal deformity, which have varied widely depending on the indication and fixation techniques used [10, 11, 18, 20, 24, 32, 34, 35, 43]. Fusion rates with minimally invasive transsacral fusion have been reported at 91% to 96% [8, 22, 46]. None of these studies however accounted for transsacral fusion in the setting of a long-segment fusion. Rather these studies report outcomes for short-segment fusions in the setting of low-grade spondylolisthesis or degenerative disc disease. It is thus difficult to compare our data to these as the clinical settings where transsacral fusion was used were quite different. Kim et al. [32] noted a pseudarthrosis rate of 24% in their series of 144 patients undergoing surgery for adult spinal deformity, with a mean followup of 2 years. They noted risk factors for pseudarthrosis to include thoracolumbar kyphosis, osteoarthritis of the hip, thoracoabdominal approach (versus paramedian approach), a positive sagittal balance of 5 cm or more at 8 weeks postoperatively, older age at surgery (> 55 years), and incomplete sacropelvic fixation. Prevention of pseudarthrosis is important because pseudarthrosis may lead to pain, loss of deformity correction, progressive deformity, or neurologic deficit [33]. Additionally, Scoliosis Research Society Outcome Instrument 24 scores have been known to be significantly lower in adult patients with pseudarthrosis than in those achieving solid fusion [33].

HRQOL data improved after this surgery. Improvements noted here compare well with normative data reported in correction of adult scoliosis. In one systematic review of 49 articles of patients undergoing scoliosis correction, ODI was noted to improve by 15.7 points [48].

Our overall complication rate was 39% and 17% of the complications were directly related to transsacral fixation. The presacral approach may have reduced risk of bowel and vascular injury when compared to ALIF. ALIF has been associated with vascular injury rates ranging from 0.5% to 15.6%, a bowel injury rate of 1.6%, and a prolonged ileus rate of 0.6% [9, 13, 40, 44]. The biggest hesitation for surgeons to perform this procedure is the possibility of bowel injury. In a review of 5300 cases of TranS1 AxiaLIF^®^ performed in the United States from January 2005 to January 2009, per the FDA medical device reporting data, the complication rate in terms of bowel injury with AxiaLIF^®^ was 0.47% and the overall complication rate was 0.7% [5]. Gundanna et al. [25] found a 1.3% overall complication rate in a retrospective analysis of 9152 patients undergoing AxiaLIF^®^. In that study, the most commonly reported complication was bowel injury (0.6%). In our experience, we have not seen any bowel or vascular injury. Additionally, we did not see any sacral insufficiency fractures or sacral screw loosening or breakage, except in cases that went on to pseudarthrosis.

We found transsacral fusion to be a safe, less invasive approach to achieving fixation and fusion across the L5-S1 disc space. In this series of 46 patients, the procedure had a low complication rate, while achieving fusion rates and clinical outcomes comparable to those of more invasive open surgeries when compared with historical controls. Our loss to followup (18%, 10 of 56 patients) may have resulted in an underestimation of the frequency of pseudarthrosis and reoperation, and our results should be interpreted in light of this. Even so, this technique may obviate the need for iliac screw fixation in selected patients, as biomechanically it provides for a strong anchor at L5-S1 while off-loading the sacral pedicle screws. In nonosteopenic patients, who have not had prior rectal surgery and do not have presacral adhesions or aberrant midline vasculature, transsacral fixation and fusion may provide a viable alternative for achieving interbody lumbosacral fusion in the setting of long-segment spinal fusion. Future studies should address fusion rates of TLIF/ALIF versus transsacral fusion in the setting of scoliosis correction in a prospective, randomized way.
